# Survey of haemoprotozoa and *Toxoplasma gondii* in little penguins in Lutruwita/Tasmania, Australia

**DOI:** 10.1016/j.ijppaw.2025.101167

**Published:** 2025-11-24

**Authors:** Melanie R. Wells, Scott Carver, Ralph Eric Thijl Vanstreels, Annie Philips, Mary-Anne Lea, Michelle Power

**Affiliations:** a Department of Biological Sciences, School of Natural Sciences, University of Tasmania, Hobart, 7001, Tasmania, Australia; b Institute for Marine and Antarctic Studies, Battery Point, 7004, Tasmania, Australia; c Centre for Marine Sociology, University of Tasmania, Hobart, 7001, Tasmania, Australia; d Odum School of Ecology, University of Georgia, Athens, 30602, GA, USA; e Center for the Ecology of Infectious Diseases, University of Georgia, Athens, 30602, GA, USA; f Karen C. Drayer Wildlife Health Center, One Health Institute, School of Veterinary Medicine, University of California, Davis, 95616, CA, USA; g Wildlife Veterinary Consultant, Hobart, 7000, Tasmania, Australia; h School of Natural Sciences, Faculty of Science and Engineering, Macquarie University, Sydney, 2109, New South Wales, Australia

**Keywords:** Blood parasites, Health assessment, *Babesia*, Wildlife health

## Abstract

Parasitism poses potential health risks to penguin populations, yet the prevalence of protozoan parasites in wild free-living populations remains poorly understood. We conducted a survey of haemoprotozoa and *Toxoplasma gondii* in little penguins (*Eudyptula minor*) across 23 colonies in Lutruwita/Tasmania, Australia. Blood samples were screened for haemoprotozoa using light microscopy and nested PCR. Suspect intraerythrocytic inclusions were seen in the blood smears of 25 % of the penguins examined (62/247), but morphological and molecular evidence only confirmed *Babesia* sp. infection in 2.4 % of penguins (6/247). A single blood smear exhibited sufficient parasite life stages to allow morphological identification, and the morphology was consistent with *Babesia peircei*. Sequencing of the *18S rRNA* gene of 4 samples confirmed a close relationship to *Babesia* sp. previously reported in little penguins in Lutruwita/Tasmania. A subset of samples (*n* = 50) with intraerythrocytic inclusions tested negative for *Haemoproteus* sp., *Leucocytozoon* sp., and *Plasmodium* sp. Antibodies against *T. gondii* were detected in 3/122 penguins, though only one sample (0.8 %) was considered seropositive (titre ≤1:64). This study provides a contemporary baseline for protozoan parasite occurrence in wild little penguins at the southernmost part of their Australian range. As changing climates are facilitating range expansion of vector species, studying the health of populations at the edge of their range is critical.

## Highlights

• Suspect intraerythrocytic inclusions observed in 25 % of penguin blood smears.
• Babesia sp. infection confirmed in 2.4 % of little penguins.
• Babesia sp. related to previously reported species from AUS and NZ.
• Antibodies against *T. gondii* detected in 2.5 % of penguins.

## Graphical abstract

**Figure:**
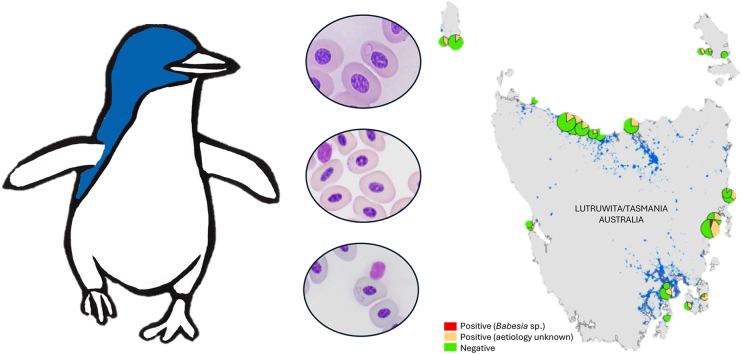

## 1 Introduction

Parasitism is an important health and conservation concern for many penguin species. In captive settings penguins are highly susceptible to infection by blood parasites (haemoprotozoa), some of which can cause a rapid disease onset and high mortality (Grilo et al., 2016; Vanstreels et al., 2016). However, the occurrence and prevalence of haemoprotozoa, and many other parasite species, in wild penguin populations is less well understood (Jones and Shellam, 1999; Vanstreels et al., 2016).

Haemoprotozoa are a diverse group of vector-borne blood parasites of vertebrate hosts. Haemoprotozoan parasites of penguins include dipteran-borne Haemosporida (*Plasmodium* spp., *Haemoproteus* spp., and *Leucocytozoon* spp.), tick-borne Piroplasmida (*Babesia* spp.), and Kinetoplastea (*Trypanosoma* spp.) for which the vectors in penguins remain unconfirmed (Vanstreels et al., 2016). Penguins are also susceptible to *Toxoplasma gondii* (Ploeg et al., 2011; Acosta et al., 2019; Campbell et al., 2022), an apicomplexan parasite spread through the excretion of environmentally robust infectious oocysts in felid faeces (Dubey et al., 2020). These oocysts are disseminated to marine systems via wastewater and can survive seawater for 6 months (Lindsay and Dubey, 2009), which can lead to exposure in marine fauna (Jensen et al., 2010; Li et al., 2022).

Significant overlap in the range of temperate penguin populations and known haemoprotozoan vectors (mosquitoes, biting midges, black flies, and ticks; Vanstreels et al., 2016), and proximity to urban wastewater runoff, potentially polluted with *T. gondii* oocysts (Lindsay and Dubey, 2009), increases risk of parasitism for penguins. Warming climates are thought to be facilitating range expansion of haemoprotozoan vector species to colder regions which facilitates dissemination of haemoprotozoa to new geographic areas (Githeko et al., 2000). For example, tick-borne *Babesia* sp*.* were detected in penguins at Vapour Col, Deception Island, Antarctica (Montero et al., 2016), a colony known to have a particularly high tick density (Barbosa et al., 2011) possibly due to slightly warmer soil microhabitat provided by volcanic-hydrothermal processes (Goyanes et al., 2014). Haemoprotozoa infections in birds are also influenced by habitat fragmentation and the presence of concurrent stressors (Perrin et al., 2023) which many penguin species across the globe are experiencing, including the little penguin (*Eudyptula minor*) in Australia (Ropert-Coudert et al., 2019).

The little penguin, the smallest penguin species, is only found in the temperate waters of Australia and Aotearoa/New Zealand. Little penguin populations persist along a spectrum of marine, terrestrial and anthropogenically modified habitats, being exposed to a range of conservation threats. The southernmost extent of little penguin range in Australia is the island state of Lutruwita/Tasmania, which is considered the population stronghold (Dann et al., 1996). Notably, the surrounding marine environment is thought to be warming four times faster than the global average, giving rise to acute marine heat wave events (Ridgway and Ling, 2023).

Haemoprotozoa were first described in little penguins in Lutruwita/Tasmania in the 1980s with the first and only observation of *Trypanosoma* infection in any penguin species (Jones and Woehler, 1989). *Babesia*, *Plasmodium,* and *Haemoproteus* have also been reported in little penguins (Harvey and Alley, 2008; Cannell et al., 2013; Vanstreels et al., 2015; Sijbranda et al., 2017a, Sijbranda et al., 2017b), but the prevalence of these parasites across their range remains unknown. The pathogenicity of the haemoprotozoan parasites for little penguins is also poorly understood. Mortality caused by toxoplasmosis (*T. gondii*) has been recorded in little penguins in captivity (Mason et al., 1991) and in West Australia (Campbell et al., 2022), but there are no data on exposure in little penguins in Lutruwita/Tasmania.

Here, we aimed to conduct a survey of potentially pathogenic haemoprotozoa in wild little penguin populations along the southernmost extent of their Australian range. A contemporary assessment of haemoprotozoa presence is critical given increasing anthropogenic pressure on the little penguin, and specifically the changing climate which may influence haemoprotozoa vector range. Using both morphological and targeted molecular techniques, we assessed the prevalence of haemoprotozoa and *T. gondii* in free-living adult little penguins in Lutruwita/Tasmania.

## 2 Screening for haemoprotozoa

**Fig. 1:**
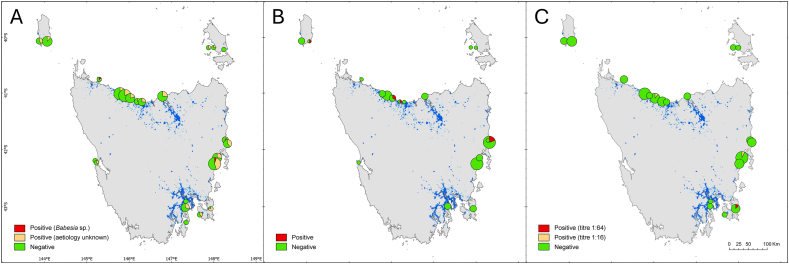
Geographic distribution of 23 little penguin colonies surveyed for haemoprotozoa and *Toxoplasma gondii* in Lutruwita/Tasmania. A) Light microscopy screening of blood smears for intraerythrocytic inclusions (*n* = 247). B) Nested PCR targeting the *18S rRNA* gene of *Babesia* sp. (*n* = 50). C) Modified agglutination test targeting antibodies against *T. gondii* (*n* = 122). Pie chart sizes drawn proportionally to sample size. Blue shaded areas represent human population density (WorldPop, 2020).

We used a combination of targeted and non-targeted diagnostic methods (Wells et al., 2023) to evaluate the occurrence of haemoprotozoa (Table 1). Blood samples were collected from 317 little penguins across 23 colonies between 2021 and 2023 (Fig. 1; see File S1 for metadata). Blood was extracted by venipuncture of the medial metatarsal vein and placed directly into an EDTA tube which was later centrifuged (10,000 RPM, 10 min) and the serum and whole blood frozen separately (−20^o^C) in polypropylene tubes. Blood smears were prepared immediately after collection and fixed with absolute methanol for 15 s at the time of collection and stained with a rapid Romanowsky stain (Diff-Quik, Medion Diagnostics GmbH, Düdingen, Germany) within 1–14 days of collection.

**Table 1:** Summary of diagnostic results for haemoprotozoa and *Toxoplasma gondii* in little penguins from Lutruwita/Tasmania, Australia. Results are summarized as “positive individuals/examined individuals”.

Colony (geographic coordinates)	Intraerythrocytic inclusions in blood smears	Molecular detection of *Babesia*^a^	Molecular detection of Haemosporida^a^	Serology for *T. gondii*
Bass Strait islands				
King Island, West (40.063°S 143.878°E)	4/10	0/3	0/3	0/5
King Island, Grassy (40.069°S 144.063°E)	2/16	1/2	0/2	0/10
East Kangaroo Island (40.182°S 147.903°E)	2/5	0/1	0/1	0/3
Big Green Island (40.183°S 147.980°E)	1/4	0/1	0/1	0/2
Fisher Island (40.217°S 148.239°E)	0/4	–	–	–
North coast				
Stanley (40.756°S 145.295°E)	2/7^b^	0/1	0/1	0/6
Doctors Rocks (41.013°S 145.785°E)	3/23	0/3	0/3	0/13
Burnie (41.045°S 145.899°E)	5/27	0/4	0/4	0/4
Sulphur Creek (41.093°S 146.028°E)	3/15	1/3	0/3	1/10^c^
Ulverstone (41.152°S 146.200°E)	2/10^b^	1/2	0/2	0/9
Lillico (41.160°S 146.303°E)	3/12	0/2	0/2	0/4
Low Head (41.058°S 146.791°E)	4/16	0/3	0/3	0/5
West coast				
Entrance Island (42.211°S 145.216°E)	0/5	–	–	–
Bonnet Island (42.223°S 145.222°E)	1/3	0/1	0/1	–
East coast				
Diamond Island (41.858°S 148.290°E)	1/10	0/1	0/1	0/6
Bicheno (41.870°S 148.296°E)	5/13	1/5	0/5	0/8
Coswell (42.145°S 148.080°E)	4/15	0/3	0/3	1/14^c^
Little Christmas Island (42.250°S 148.019°E)	9/20^b^	0/9	0/9	0/9
Red Chapel (42.906°S 147.342°E)	0/4	–	–	0/1
Boronia (42.990°S 147.329°E)	4/13	0/3	0/3	0/3
Pirates Bay (43.032°S 147.932°E)	4/5	0/3	0/3	1/8^d^
Wedge Island (43.135°S 147.672°E)	3/8	–	–	0/2
Bruny Island (43.271°S 147.348°E)	0/2	–	–	–

**Total**	62/247	4/50	0/50	3/122

**Fig. 2:**
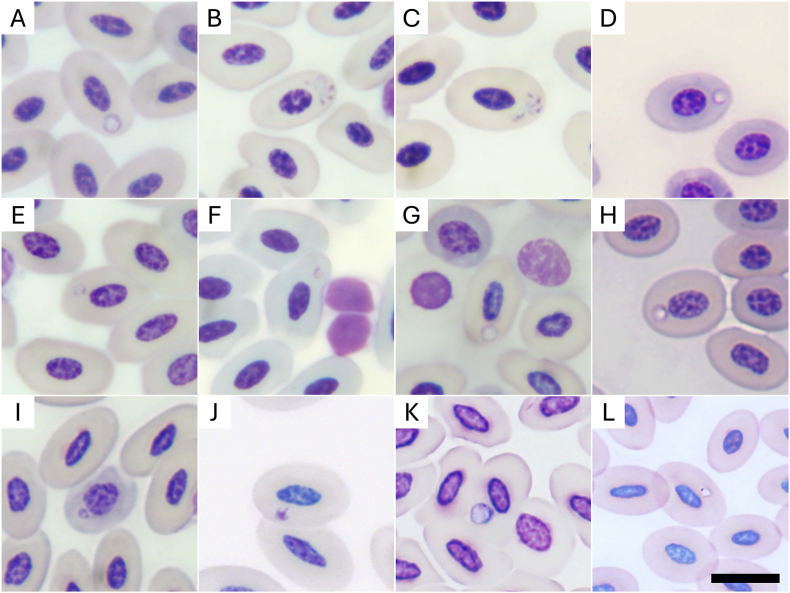
Intraerythrocytic inclusions in blood smears from little penguin. A–D: Inclusions considered unequivocally consistent with *Babesia* sp., even though frozen blood samples were not available for molecular testing. E–H: Inclusions seen in individuals that were confirmed as PCR-positive for *Babesia* sp. I–L: Inclusions seen in individuals that tested negative in PCR tests for *Babesia*, *Haemoproteus*, *Plasmodium*, and *Leucocytozoon*. Sample identification: A–C: LCI40, D: ST46, E: PT43, F: UW10, G: SC40, H: GR01, I: BN41, J: LCI20, K: PT45, L: DR43. Diff-Quik stain. Scale-bar = 10 μm.

Notes.

a Subset of samples from penguins where intraerythrocytic inclusions were seen in blood smears.

b Intraerythrocytic inclusions of one individual were determined to be *Babesia* sp. on the basis of morphology (see Fig. 2A–F).

c Antibody titre 1:16.

d Antibody titre 1:64.

Of the 317 individuals sampled, 70 of the blood smears were unable to be read due to poor staining (*n* = 39), damage to the slide (*n* = 3), insufficient (*n* = 13) or excessive volume of blood (*n* = 15). While the attrition in samples is unfortunate (70/317; 22.1 %), these uncountable slides were spread across 18 of the 23 sites, therefore we do not suspect any colony-specific bias in our prevalence estimates. Most of the unreadable slides (63/70; 90 %) were collected early during the overall sampling period and therefore are likely a reflection of staining and technique inexperience. The remaining slides (*n* = 247) were screened for the presence of haemoprotozoa under light microscopy for a minimum 20 min. Small intraerythrocytic inclusions considered suspect for haemoprotozoa were seen in 62 blood smears (25.1 %; Fig. 1 and Table 1). One of the inclusion-positive blood smears showed a combination of round forms (“rings”, Fig. 2A), amoeboid forms (“cow's udder”, Fig. 2B), and tetrad meronts with distally-positioned chromatin (Fig. 2C); these characteristics are consistent with *Babesia peircei*, described from African penguins (*Spheniscus demersus*) in South Africa (Earlé et al., 1993). Another inclusion-positive blood smear had structures unequivocally consistent with *Babesia* sp. round forms (Fig. 2D). The remaining inclusion-positive blood smears had poorly-defined structures that precluded their aetiology from being confidently determined (Fig. 2E–L). No extracellular parasites (e.g. *Trypanosoma* sp., microfilariae) or spirochaetes (e.g. *Borrelia* sp.) were seen.

The primary intention for the blood smear examination was a differential white cell count of 100 leucocytes (immersion oil, 1000 × ), collected as part of a PhD thesis, so the parasite assessment was somewhat opportunistic; hence we unfortunately lacked standardisation in erythrocyte counts. The total number of fields examined varied between 20 and 458 (median = 65). However, based on data from a subset of these individuals (*n* = 45) used in a different study (Wells et al., 2024), the average number of erythrocytes per field was 190.36 (range = 105–250), therefore the estimated average number of erythrocytes examined per sample was 14,668 (range = 3807–87,185; median = 12,373). Although this lack of standardisation would evidently result in a detection bias, we still detected intraerythrocytic inclusions in 26.8 % of the smears where <10,000 erythrocytes were examined. Nonetheless, future studies should aim to reduce the probability of detection bias, as well as include frequency estimates.

Of the 62 penguins with suspect intraerythrocytic inclusions in the blood smears, 50 had a corresponding sample of frozen red blood cells for DNA extraction for molecular screening (Table 1); unfortunately, these samples did not include the two penguins for which inclusions had been confidently assigned to *Babesia* sp. as mentioned above. DNA was extracted using DNeasy Blood and Tissue Kits (QIAGEN, Valencia, CA), following the manufacturer's instructions. DNA extraction was verified with a polymerase chain reaction (PCR) targeting the *18S rDNA* universal Eukaryote gene (Hadziavdic et al., 2014). Nested PCR assays were used to amplify segments of the mitochondrial cytochrome *b* (*cyt-b*) gene of *Haemoproteus*/*Plasmodium* or *Leucocytozoon* (Hellgren et al., 2004) and a segment of the 18S ribosomal RNA (*18S rRNA*) gene of *Babesia* (Vanstreels et al., 2015). To ensure optimal amplification of the control samples, the annealing temperature was increased to 54 °C. Amplification products were visualized with Green GoTaq (Promega, Australia) in 2 % agar gel electrophoresis. Blood samples from Taronga Zoo infected with *Plasmodium relictum* and *Leucocytozoon* sp. (Sample ID 60351, 15001, and 70353) and DNA-free water were used as positive and negative controls, respectively. All samples were negative for *Haemoproteus*/*Plasmodium* and *Leucocytozoon*. Four samples were positive for *Babesia* sp. Re-examination of the blood smears from these individuals identified intraerythrocytic inclusions that were not well defined but could be reasonably considered consistent with *Babesia* sp. (Fig. 2E–H).

PCR-positive samples were subjected to Sanger sequencing of the *18S rRNA* gene at a commercial laboratory (Ramaciotti Centre for Genomics, Sydney, Australia) and resulting sequences were deposited in GenBank (accession codes PX232659–62). BLAST searches (https://www.ncbi.nlm.nih.gov/geo/query/blast.html) revealed that *18S rRNA* sequences from all four samples were 100 % identical to those of *Babesia* sp. previously detected in little penguins in Lutruwita/Tasmania (accession codes KP144322–3; Vanstreels et al., 2015). Reference piroplasmid *18S rRNA* sequences were aligned with MAFFT v7.52 (Katoh and Standley, 2013), and then IQ-TREE v2.2.2.6 (Minh et al., 2020) was used to construct a maximum likelihood tree with the GTR + I + G4+F nucleotide evolution model and 1000 ultrafast bootstrap replicates (Fig. S1). This analysis revealed that the sequences from little penguins in Lutruwita/Tasmania were part of the “*Peircei* group” (clade V *sensu* Jalovecka et al., 2019), clustering with *Babesia* sp. previously detected from chinstrap penguins (*Pygoscelis antarcticus*) in Antarctica (Montero et al., 2016), and from other seabirds in New Zealand (Paparini et al., 2014).

Because of the relatively low proportion of samples that had suspect intraerythrocytic inclusions on blood smears but which yielded positive PCR results for *Babesia* sp. (8 %; 4/50), we re-tested extracted DNA samples with a different PCR protocol, targeting a different segment of the *18S rRNA* gene of *Babesia* sp. (Jefferies et al., 2007). This second PCR protocol yielded identical results, with only the same four samples identified as positive. We chose nested PCR protocols as a widely used, cost-effective method and due the increased sensitivity and specificity for detecting mixed Haemosporidian infection, common in birds (Hellgren et al., 2004; Musa et al., 2024). We do acknowledge, however, that in cases of low infection, real-time PCR may be more sensitive at detecting prevalence (Sijbranda et al., 2017a, Sijbranda et al., 2017b; Ishtiaq et al., 2017). While we cannot completely rule out false negatives of low intensity infections, or that problems with DNA amplification (e.g. poor primer annealing) may have interfered with our PCRs to detect haemoprotozoan DNA, it seems reasonable to suspect that many of the intraerythrocytic inclusions seen in the blood smears had a different aetiology, which should be explored in future studies. These observations could correspond to infectious agents that we did not test for, such as *Anaplasma* sp. or other *Rickettsiales* bacteria (Parsons et al., 2018; Vanstreels et al., 2018), or they may represent cellular abnormalities resulting from exposure to toxic substances (Gómez-Meda et al., 2006; Harr et al., 2017; Wells et al., 2024). It is also possible that these inclusions resulted from methodological artefacts (e.g. stain aggregates, haemoglobin crystals) or natural processes (nuclear fragmentation, retention of cytoplasmic organelles), representing pseudo-parasites with little health significance. Of note, during an earlier survey of haemoprotozoan parasites in little penguins in Australia (Vanstreels et al., 2015) it was also noted that intraerythrocytic inclusions were relatively frequent in this species’ blood smears; at the time, most of these structures were dismissed as pseudo-parasites, with only a small subset being considered consistent with *Babesia* sp. (R.E.T. Vanstreels, pers. obs.). Nonetheless, further research should seek to clarify the aetiology and any associated health perturbances of these intraerythrocytic inclusions, through targeted PCR for possible alternative haemoprotozoa or the use of higher-resolution microscopic imaging. Additionally, including a frequency of occurrence count will help to understand apparent infection intensity. Although all individuals sampled in the present study were exhibiting natural behaviours, future research should also focus on identifying sublethal health perturbances potentially resulting from intraerythrocytic inclusions.

Neither the intraerythrocytic inclusions and the confirmed *Babesia* sp. detections (both through PCR and microscopy) presented any patterns in colony-level detection frequency or obvious geographic clustering (Table 1 and Fig. 1A and B). However, there appeared to be higher proportions of intraerythrocytic inclusions coinciding with samples from late summer and moulting birds (both typically occurring at the same time; File S1). Not only does seasonality positively correlate with ectoparasite load on little penguins, but moulting birds are known to be particularly susceptible to ectoparasite infection (Wells et al., 2025). We additionally explored for colony-level detection frequencies for the intraerythrocytic inclusions, which was generally consistent across colonies (mean and median detection rate of 25 %), except for the Pirate's Bay colony, which had a relatively high detection rate of 80 % [CI 0.37, 0.96], though an overall low sample size (*n* = 5). Ongoing investigations should seek to further explore this observation and target future sampling. If we only consider as positive the four PCR-positive penguins and the two penguins with intraerythrocytic inclusions that were unequivocally consistent with *Babesia* sp. but for which no samples were available for PCR testing (Fig. 2A–D), the apparent prevalence of *Babesia* sp. in this study was 2.4 % (6/247). An earlier survey had detected *Babesia* sp. in the blood smears of 2.8 % of little penguins (*n* = 141) sampled in Lutruwita/Tasmania in 2012–2013 (Vanstreels et al., 2015), suggesting that the apparent prevalence of these parasites has not changed drastically over the last decade. Of note, only two penguins with confirmed *Babesia* sp. infections (33 %) had ticks on external examination (*cf*. overall tick prevalence 30.8 %; File S1), which highlights that tick presence at the time of sampling is not a reliable predictor of *Babesia* infection status.

## 3 Screening for *Toxoplasma gondii*

For *T. gondii*, serological screening was performed for 122 individuals for which sufficient blood volume was collected (Table 1). Plasma samples were analysed at the Tasmanian Department of Natural Resources and Environment's Animal Health Laboratory using a modified agglutination test (MAT) (Liyanage et al., 2024). Though this has not specifically been validated for little penguins, it is a universally used method, and validation in cats revealed test specificity of 96 % (Adriaanse et al., 2020). Of the three reactive samples (2.5 %), one had an antibody titre of 1:64 (0.8 %), the other two samples were below the cut-off with a titre of 1:16. Interpretation of MAT tests considers that only antibody titres equal to or greater than 1:25 or 1:64 are indicative of *T. gondii* infection (Dubey, 2008; Fancourt and Jackson, 2014). In this context, the apparent prevalence of *T. gondii* in this study would be considered 0.8 % (i.e. one positive penguin). Nevertheless, understanding antibody dynamics in response to infection can be difficult from a cross-sectional sampling regime (Barbosa and Palacios, 2009), and titres below the cut-off can still be relevant as they could represent early stages of infection or reflect past exposures.

There was no apparent geographic clustering of the reactive individuals, nor any obvious association with areas of high human population density (Fig. 1C). Given that Tasmania does not have native felids, cats (domestic, stray, or feral) must have been the source of *T. gondii* oocysts to which the penguins were exposed, whether directly (exposure to cat faeces) or indirectly (ingestion of infected prey; Dubey, 2008). Past studies have shown that >80 % of stray and feral cats in Lutruwita/Tasmania are seropositive to *T. gondii* and presumably represent a major source of environmental contamination with oocysts (Fancourt and Jackson, 2014). Considering that *T. gondii* can cause rapid and lethal infections to penguins (Mason et al., 1991; Ploeg et al., 2011; Campbell et al., 2022) and some Australian marsupials (Cranfield et al., 1990; Dubey et al., 2021), our results highlight this parasite as a possible conservation concern. Further studies are therefore warranted to better understand the ecology of this parasite in the region and its potential health impacts on penguins and other native wildlife.

## 4 Conclusions

This study has documented the occurrence of *Babesia* sp. and the exposure to *T. gondii* in wild little penguins around Lutruwita/Tasmania. Although the study relied on samples from wild free-living penguins, exhibiting natural behaviours, these parasites have been previously demonstrated to be pathogenic to little penguins, with potentially lethal outcomes (Mason et al., 1991; Campbell et al., 2022). Unfortunately, we are unable to infer causative clinical health implications from our study design and longitudinal studies are required to understand health effects on fitness or survival. Though we know little penguins can be somewhat resilient to health effects from ectoparasitism (Wells et al., 2025), future research should target the sublethal effects that protozoa parasites may present. The investigation of the causes of mortality of little penguins through necropsies and targeted surveillance of protozoa in tissue samples, as has been done for little penguins in West Australia (Cannell et al., 2013; Campbell et al., 2022) and Aotearoa/New Zealand (Saverimuttu et al., 2025), would be valuable to shed light on the potential impacts of these parasites in Lutruwita/Tasmania. Our results also revealed that little penguins in Lutruwita/Tasmania have a relatively high prevalence (25 %) of intraerythrocytic inclusions for which the aetiology is unknown, warranting further research to better understand aetiology through targeted PCR or as initial step, improved microscopic imaging capability. In the case of *T. gondii*, additional research on the routes of exposure of little penguins to this cat-borne parasite could be valuable to understand how its potential impacts on this and other native species may be mitigated. Climate change is expected to facilitate range expansion of protozoal parasites and their vectors (Süss et al., 2008; Short et al., 2017). In this context, because it represents the southernmost extent of the little penguin distribution in Australia, Lutruwita/Tasmania should be considered a key area for monitoring how the species and its parasites will respond to a changing climate.

## CRediT authorship contribution statement

**Melanie R. Wells:** Writing – review & editing, Writing – original draft, Methodology, Investigation, Funding acquisition, Formal analysis, Data curation, Conceptualization. **Scott Carver:** Writing – review & editing, Writing – original draft, Supervision, Project administration, Funding acquisition, Formal analysis, Conceptualization. **Ralph Eric Thijl Vanstreels:** Writing – review & editing, Writing – original draft, Methodology, Formal analysis, Conceptualization. **Annie Philips:** Writing – review & editing, Writing – original draft, Methodology, Funding acquisition, Conceptualization. **Mary-Anne Lea:** Writing – review & editing, Writing – original draft, Project administration, Funding acquisition, Conceptualization. **Michelle Power:** Writing – review & editing, Writing – original draft, Supervision, Resources, Methodology, Investigation, Funding acquisition, Formal analysis.

## Data availability statement

All raw data associated with this study is submitted as supplementary material.

## Animal ethics and permits

This study was conducted in accordance with the University of Tasmania's Animal Ethics Committee approval (Project: 23764) and the Tasmanian Department of Natural Resources and Environment Scientific Permits (Fauna) (Authority numbers: FA23016, FA21015 and FA22262).

## Funding

This research was funded by The 10.13039/501100001160Australian Society for Parasitology through a researcher exchange. Fieldwork was supported by the 10.13039/501100008702Ecological Society of Australia through the Holsworth Wildlife Endowment, the 10.13039/501100000969Australian Academy of Science through the Margaret Middleton Fund for Endangered Australian Native Vertebrate, and student support grants from the 10.13039/100026856Wildlife Disease Association of Australasia and the Australasian Seabird Group. Mel Wells was supported by a Research Training Program Stipend Scholarship provided to the 10.13039/501100001249University of Tasmania by the 10.13039/100015539Australian Government.

## Conflict of interest

The authors declare no conflict of interest.

## Appendix A Supplementary data

Fig. S1 Maximum likelihood tree for the *18S rRNA* gene of piroplasmids. Colours are used to highlight parasites from this study (red) and from other avian hosts (blue). Strains are grouped as per Jalovecka et al. (2019). Branch lengths are drawn proportionally to evolutionary distance (scale-bar). Values near nodes represent ultrafast bootstrap values. Node tips are labelled with GenBank accession code, parasite species, host species, and location (avian hosts only). Fig. S1
