# Supplementary figures and images for: Survey of haemoprotozoa and Toxoplasma gondii in little penguins in Lutruwita/Tasmania, Australia
Source: Int J Parasitol Parasites Wildl. 2025 Nov 24;28:101167. doi: 10.1016/j.ijppaw.2025.101167 (PMC12702176; doi:10.1016/j.ijppaw.2025.101167)

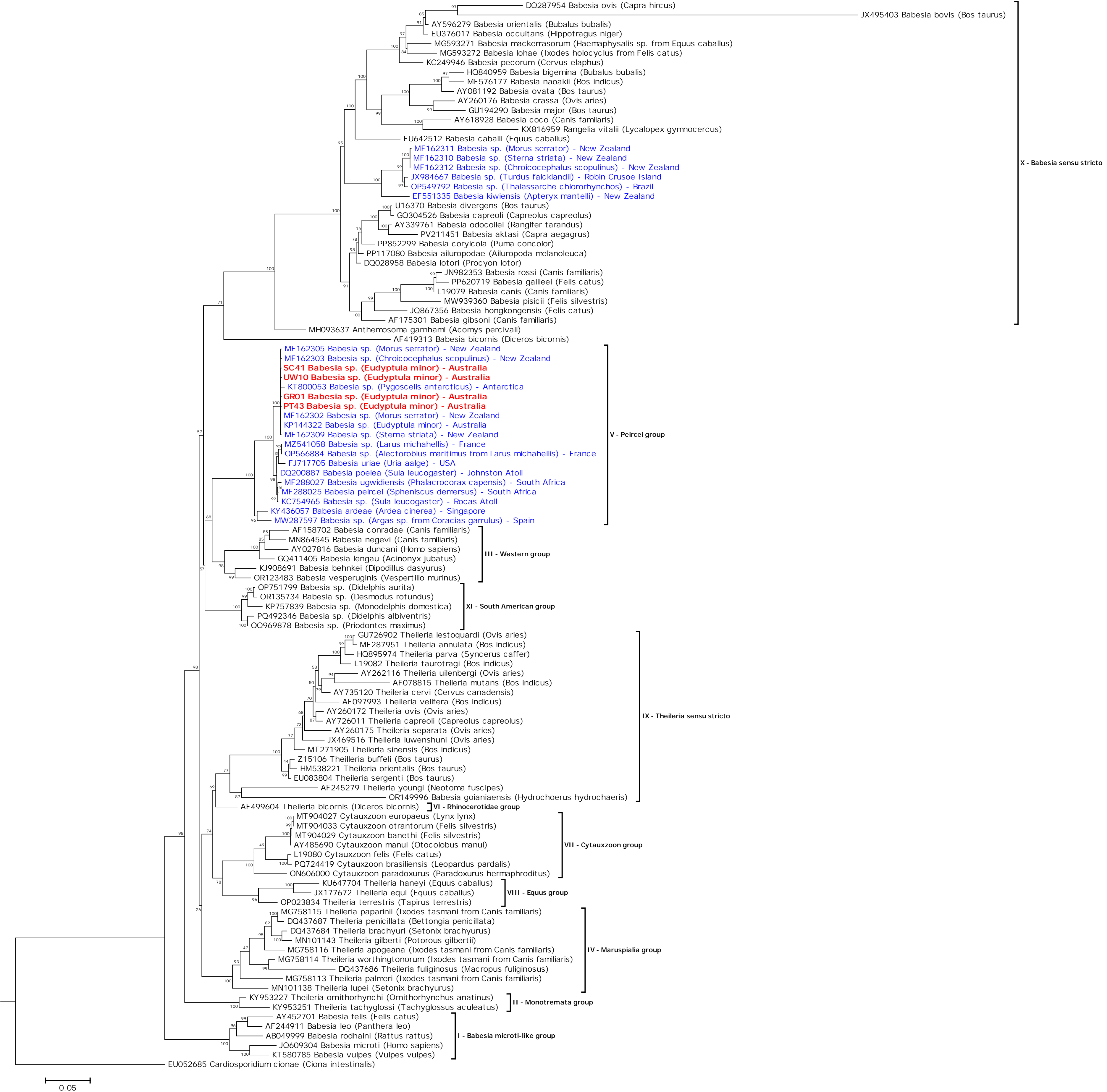

Supplement: Fig. S1 — Maximum likelihood tree for the 18S rRNA gene of piroplasmids. Colours are used to highlight parasites from this study (red) and from other avian hosts (blue). Strains are grouped as per Jalovecka et al. (2019). Branch lengths are drawn proportionally to evolutionary distance (scale-bar). Values near nodes represent ultrafast bootstrap values. Node tips are labelled with GenBank accession code, parasite species, host species, and location (avian hosts only). [file mmc1.pdf]
